# Supplementary material for: The status of prehospital care delivery for COVID-19 patients in Addis Ababa, Ethiopia: The study emphasizing adverse events occurring in prehospital transport and associated factors
Source: PLoS One. 2022 Feb 1;17(2):e0263278. doi: 10.1371/journal.pone.0263278 (PMC8806066; doi:10.1371/journal.pone.0263278)
Supplement: S1 File — (DOCX) [file pone.0263278.s001.docx]

**Data collection tool**

Are you willing to let the information to be used for this study?

1. Yes 2. No

Name of the COVID-19 treatment center_________________

Name of the data collector____________________

Supervisor Name___________________________

Date:___________________

Code given_________________

**Section one: Socio-demographic and clinical characteristics**

| **S.N** | **Variable** | **Description** | **Skip** |
| --- | --- | --- | --- |
| **1** | Age of the patient in years |  |  |
| **2** | Sex | 1. Male  2. Female |  |
| **3** | Qualification of transporting personnel | 1. EMT 2. Clinical nurse 3. BSc nurse/general 4. BSc/ECC nurse 5. Health officer 6. GP 7. Other (specify) |  |
| **4** | EMS experience of transporting personnel (in months) |  |  |
| **5** | Time at which transport begun |  |  |
| **6** | Time at which the patient reach receiving facility |  |  |
| **7** | Total pre hospital time in minutes________________ |  |  |
| **8** | Time at which the patient developed adverse events (only if the patient developed it) |  |  |
| **9** | Overall distance travelled (in KM) |  |  |
| **10** | Vital sign before transport | 1. Temperature_____^o^C 2. BP_________mmhg 3. PR__________/min 4. RR__________/min 5. Oxygen saturation_____% 6. Not taken |  |
| **11** | Vital sign after transport | 1. Temperature_____^o^C 2. BP_________mmhg 3. PR__________/min 4. RR__________/min 5. Oxygen saturation_____% 6. Not taken |  |
| **12** | Presence of Comorbid illness | 1.Yes  2. No | **If no skip to 13** |
| **13** | Type of comorbid illness | 1. DM 2. Hypertension 3. Chronic heart disease 4. Renal disease 5. Malignancy 6. Asthmatic 7. COPD 8. HIV/AIDS 9. Other (Specify)   __________________ |  |
| **14** | Severity of COVID-19 (before transportation) | 1. Mild 2. Moderate 3. Severe 4. Critical 5. Not identified |  |
| **15** | Severity of COVID-19 (after transportation) | 1. Mild 2. Moderate 3. Severe 4. Critical 5. Not identified |  |
| **16** | Medication given to the patient during transport | _____________________ |  |

**Section two: The Pittsburgh Adverse Events Detection and Classification Tool (PittAETool)**

**Step 1: Triggers**

| S.N | ﻿**Triggers** | Yes | No |
| --- | --- | --- | --- |
| 1 | **Documentation Triggers** |  |  |
|  | ﻿Missing, incomplete, or unclear documentation for the following: chief complaint, physical assessment, vital signs, hemodynamic monitoring (e.g., ETC02), allergies, pertinent history or medications, patient condition at handoff of facility. |  |  |
|  | ﻿**Operational & Patient Movement Triggers** |  |  |
| 2 | ﻿Time from initial patient contact to transfer of care exceeds accepted standards |  |  |
| 3 | ﻿Injury to patient or team member during patient encounter / transport (e.g., stretcher drop, needle stick, or other) |  |  |
| 4 | ﻿Request for additional resources, personnel, or supervisor due to change in patient condition |  |  |
|  | ﻿**Patient Condition Triggers** |  |  |
| 5 | ﻿A worsening trend (deterioration) in patient hemodynamic or mental status indicators (e.g. vital signs, LOC, GCS score). |  |  |
| 6 | ﻿Cardiac arrest during transport |  |  |
|  | ﻿**Intervention & Medication Triggers** |  |  |
| 7 | ﻿Use of any of the following interventions during patient care: (cardioversion, defibrillation, transcutaneous pacing, advanced airway attempt, surgical airway, Intraosseous (IO), chest decompression, chest tube). |  |  |
| 8 | Failure of any intervention or procedure during patient care (some examples include: inability to obtain vascular access after a reasonable amount of time or number of attempts, failed IO, failed Nasogastric Tube (NG) placement, failed Foley placement, failed cardioversion, failed defibrillation, failed transcutaneous pacing, failed advanced airway or rescue airway, failed surgical airway, failed chest decompression). |  |  |
| 9 | Use of following medications or fluids: (blood products, vasopressors or inotrope [e.g., dobutamine, dopamine], RSI medications [e.g., succinylcholine]) |  |  |
| 10 | Suggestive evidence of deviation from standard of care by performing an intervention or administering a medication that appears to be outside of protocol, or failure to perform an intervention or provide a medication that is within the standard of care |  |  |
| 11 | Medication error (e.g., administering wrong or unapproved dose, administering wrong or unapproved medication, administering medication via wrong or unapproved route). |  |  |

**Step 2: Proximal factors for adverse events**

| SN | **Variables** | Yes | No |
| --- | --- | --- | --- |
| 1 | **Patient related action** |  |  |
|  | Patient refused transport to a specialty care facility |  |  |
|  | Patient refused treatment specified in protocol |  |  |
|  | Patient with capacity to discontinues ongoing therapy |  |  |
|  | Patient took action that results or may result in harm to themselves or others |  |  |
| 2 | **Provider related actions** |  |  |
|  | Medication error |  |  |
|  | Procedural error |  |  |
|  | Failure to confirm order from medical command |  |  |
|  | Failure to treat pain as indicated on protocol |  |  |
|  | Documentation error |  |  |
|  | Inability to establish vascular access after a reasonable amount of time or number of attempts |  |  |
|  | Failure to administer oxygen to hypoxic patient |  |  |
|  | Failure to physically or chemically restrain a patient that is perceived to be at risk of harm to themselves or the crew |  |  |
|  | Delay in patient care due to delays by the referring or receiving facility |  |  |
|  | Patient handling mishaps |  |  |
| 3 | **Medical or Vehicle Equipment** |  |  |
|  | Suctioning device malfunctioned during use |  |  |
|  | An unanticipated malfunction with transport vehicle |  |  |
|  | Oxygen supply diminished during transport |  |  |
|  | All or any stretcher drop, tip, or malfunction |  |  |
|  | Fluid or medication pump failure or malfunction |  |  |
|  | Failure of cardiac monitor |  |  |
|  | Failure to provide care due lack of PPE |  |  |
|  | Missing equipment that was needed for use (if yes, specify the equipment)   1. ______________________________ 2. ______________________________ 3. ______________________________ 4. ______________________________ 5. ______________________________ 6. ______________________________ |  |  |
|  | Delay in transport due to patients weight |  |  |
|  | Delay in transport due to lack of appropriate equipment (non-ems). If yes, list the equipment  a)__________________  b)__________________  c)__________________  d)___________________  e)____________________ |  |  |
| 4 | **Environmental Factors** |  |  |
|  | Delay in prolonged arrival to hospital due to distance from treatment center  (specify in KM_______) |  |  |
|  | Delay in patient transport due to weather (eg. rainy weather) |  |  |
|  | Delay in patient transport due to scarcity of ambulance |  |  |
|  | Delay in identifying patient’s COVID-19 status due to lack of access to laboratory |  |  |

**Step three: rating of adverse event severity**

|  | **Rate Severity of Adverse Events** | Yes | No |
| --- | --- | --- | --- |
| 1 | **No adverse events**  Definition: A case where a Trigger was selected (e.g., cardiac arrest during transport), but no AE identified after full review |  |  |
| (A) | Cardiac arrest during transport, but all documentation supports the crews committed no error and followed protocol(s) as prescribed |  |  |
| (B) | Trigger selected due to administration of a medication. However, the use of administered medication was indicated for the patient (e.g., the crewmembers did not overdose the patient) |  |  |
| (C) | The time to transport the patient was delayed for necessary care, diagnostic procedures, or interventions outside control of medical crew (e.g. the patient was in the CT scanner at the referring facility) |  |  |
| (D) | Missing, incomplete, or unclear documentation |  |  |
| (E) | Specify other reason for your rate ‘no adverse event found’ |  |  |
| 2 | **Adverse events present-potential for harm**  Definition: An action that may lead to injury or harm but there is NO evidence that an injury or harm occurred |  |  |
| (A) | A medication error in which one type of medication was administered to a patient in place of more appropriate medication or medication listed as the standard of care (off- line protocols). |  |  |
| (B) | Administration of 500cc of saline when 300cc bolus was ordered by medical oversight |  |  |
| (C) | Failure to check vital signs before and after medication administration |  |  |
| (D) | Not adequately protecting patient’s airway. For example, no airway adjunct utilized for an unconscious patient with normal vital signs and no evidence of desaturation |  |  |
| (E) | Stretcher dropped or tipped, but no evidence the patient or crew member(s) were injured |  |  |
| (F) | Immediately recognized and quickly corrected missed intubation with no evidence of patient deterioration |  |  |
| (G) | Failure to control hemorrhage in a hemodynamically stable COVID-19 patient |  |  |
| (H) | Failure to administer ASA for a COVID-19 patient with chest pain for suspected cardiac etiology |  |  |
| (I) | Any other wrong procedure or action without clinical deterioration of the patient condition (specify) |  |  |
| (J) | Omission of any other essential procedure but without clinical deterioration of the patient condition (Specify) |  |  |
| 3 | **Adverse events presents - Harm identified** |  |  |
|  | Definition: An action or omission that led to injury or harm regardless of severity |  |  |
| (A) | Delay in recognition of missed intubation with evidence of patient deterioration; or failure to control airway within a reasonable amount of time or number of attempts with evidence of patient deterioration |  |  |
| (B) | The patient lost spontaneous respirations after drug over dosage or other procedure |  |  |
| (C) | Stretcher drop with injury to patient |  |  |
| (D) | Failure to administer eclamptic COVID-19 patient Magnesium Sulfate and then seizes for a period of time |  |  |
| (E) | A COVID-19 patient with a GCS ≤8 and evidence of respiratory compromise (i.e., SPO2 <90) but no airway intervention by crewmembers |  |  |
| (F) | Crewmembers fail to activate specialty team where indicated (Eg. CPR team) |  |  |
| (G) | Failure to control hemorrhage in a hemodynamically unstable COVID-19 patient |  |  |
| (H) | Any other wrong procedure or action with clinical deterioration of patient condition (specify)  ________________________________________________________ |  |  |
| (I) | Omission of any other essential procedure with clinical deterioration of the patient condition (Specify)  ______________________________________________________  ______________________________________________________ |  |  |
