# Supplementary material for: The status of prehospital care delivery for COVID-19 patients in Addis Ababa, Ethiopia: The study emphasizing adverse events occurring in prehospital transport and associated factors
Source: PLoS One. 2022 Feb 1;17(2):e0263278. doi: 10.1371/journal.pone.0263278 (PMC8806066; doi:10.1371/journal.pone.0263278)
Supplement: Attachment — Submitted filename: Responses to reviewers.docx [file pone.0263278.s003.docx]

**Responses to editor and reviewers**

Dear academic editor and reviewers,

Thank you for your comments and suggestions, which have crucial contribution to the manuscript. We have incorporated the comments and the details are given here.

**Response to editor**

1. The revised manuscript is corrected according to PLOS ONE requirements
2. The survey questionnaire is attached as supporting information
3. We were requested by editor to provide detail information on validation of the tool and it is included under data quality assurance part of the methods section in the revised manuscript
4. We were requested to provide IRB approval number and amend ethical statements and we did it accordingly

**Response to reviewer 1**

1. We were requested to provide further explanation on the type of EMS involved under introduction part and we did it accordingly.
2. Based on comments given by reviewer one, we modified table 1, 2 and 4, and marked the change made into the tables.
3. We are requested to include generalizability of our findings and we included into the revised manuscript.

**Response to reviewer 2**

1. The reviewer suggested on the further development of the study. Based on the suggestion, we recognized limitations of the study and suggested bootstrap simulation, and case-control study with propensity score matching for the further development of the study.
