# Supplementary material for: The status of prehospital care delivery for COVID-19 patients in Addis Ababa, Ethiopia: The study emphasizing adverse events occurring in prehospital transport and associated factors
Source: PLoS One. 2022 Feb 1;17(2):e0263278. doi: 10.1371/journal.pone.0263278 (PMC8806066; doi:10.1371/journal.pone.0263278)
Supplement: Attachment — Submitted filename: Responses to editor and reviewers.docx [file pone.0263278.s004.docx]

**Responses to editor and reviewers**

Dear academic editor and reviewers,

Thank you for your comments and suggestions, which have crucial contribution to the manuscript. We have incorporated the comments and the details are given here.

**Response to editor**

1. The manuscript is in line with PLOS ONE requirements
2. Reference list is complete and correct

**Response to reviewer 1**

1. We were requested to provide detailed explanations on the generalizability of the study findings and we did it accordingly.
